# Peripheral Inflammatory Factors and Acute Myocardial Infarction Risk: A Mendelian Randomization Study

**DOI:** 10.5334/gh.1269

**Published:** 2023-10-06

**Authors:** Yaxi Chen, Ling Zeng

**Affiliations:** 1 Department of Critical Care Medicine, West China Hospital, Sichuan University, China; 2 West China School of Nursing, Sichuan University, Chengdu 610000, Sichuan Province, China

**Keywords:** Inflammation, Interleukin-10, Interleukin-18, Mendelian randomization, Myocardial infarction

## Abstract

**Background:**

Previous observational studies have confirmed the relationship between inflammation and acute myocardial infarction (AMI), but genetic evidence is still lacking. The aim of this study was to explore the bidirectional association of multiple peripheral inflammatory factors with this disease at the genetic level.

**Methods:**

Summary data for AMI and several peripheral inflammatory factors (such as interleukin-10 and interleukin-18) were collected from published genome-wide correlation studies. Based on the correlation, independence, and exclusivity assumptions, a total of 9 to 110 instrumental variables were selected from these summary data to predict the above traits. Two-sample Mendelian randomization methods, including inverse-variance weighted (IVW), were used to make causal inferences between exposures and outcomes. Sensitivity analyses including Cochran’s Q, MR-Egger intercept, leave-one-out, forest plot, and MR-PRESSO were adopted to assess heterogeneity and horizontal pleiotropy.

**Results:**

The IVW reported that elevated peripheral levels of interleukin-10 and interleukin-18 were nominally associated with a reduced risk of AMI (OR = 0.876, 95% CI = 0.788 ~ 0.974, P = 0.015; OR = 0.934, 95% CI = 0.875 ~ 0.997, P = 0.040). The IVW also reported that the risk of AMI nominally increased the peripheral level of interleukin-10 (OR = 1.062, 95% CI = 1.003 ~ 1.124, P = 0.040). No significant heterogeneity or horizontal pleiotropy were found by sensitivity analyses.

**Conclusion:**

Both interleukin-10 and interleukin-18 were peripheral inflammatory factors genetically associated with AMI. In particular, combined with previous knowledge, interleukin-10 may have a protective effect on the onset, progression, and prognosis of the disease.

## 1. Introduction

Acute myocardial infarction (AMI) is a fatal type of coronary heart disease. It affects approximately 1.5 million people in the United States each year [1]. In China, there are over 500,000 new cases each year, and the incidence is still rising rapidly [2]. It is well known that inflammation plays an important role in the onset, progression, and prognosis of AMI [3]. Briefly, most cases of AMI are caused by the rupture of coronary atheromatous plaques and subsequent disruption of the blood supply, and the formation of atheromatous plaques is inextricably linked with inflammation [4]. After the onset of the disease, inflammation is also involved in a number of pathophysiological processes in ischemic and infarcted myocardial tissue and significantly affects the prognosis of patients [5]. Therefore, exploring the interrelationship between AMI and inflammation helps us understand the pathogenesis and evolution of AMI, and is important for the effective prevention and treatment of the disease.

Previous observational studies have found many peripheral inflammatory factors and inflammatory cells associated with AMI. Liu et al. reported that C-reactive protein (CRP) may be a prospective predictor of prognosis in patients with AMI undergoing percutaneous coronary intervention [6]. Several studies suggested that interleukin (IL)-1, IL-6 and IL-18 were associated with the onset of AMI, myocardial repair, the development of complications, and the readmission of patients to the hospital, whereas IL-10 may be antagonistic to IL-6 in this disease [7,8,9,10]. Some experiments revealed that regulation of neutrophil and monocyte function can affect the risk of sudden death after AMI, and the disease can also induce trafficking of blood lymphocytes to the bone marrow, leading to lymphopenia [11,12].

In addition, multiple inflammatory pathways are involved in the pathogenesis of AMI. Maracle et al. reported that the NF-κB pathway in the microvessels of atherosclerotic lesions was related to inflammation, atheromatous plaque morphology, and AMI [13]. Zhang et al. concluded that inflammatory pathways such as NLRP3/caspase-1, TLR4/MyD88/NF-κB, JAK/STAT, and PI3K/Akt may be potential therapeutic targets for cardioprotection and cardiac repair after AMI [14]. More importantly, the inflammatory factors and inflammatory cells mentioned above should be modulators or effectors of these inflammatory pathways. Studying these factors and cells may help increase our understanding of these pathways as well as the inflammatory mechanisms behind them.

At present, almost all findings on inflammatory factors and inflammatory cells in AMI come from observational studies and basic experiments. However, observational studies are susceptible to confounding factors, and the results of basic experiments need to be validated in a population. Thus, the above results do not provide reliable information. A Mendelian randomization (MR) study is regarded as a genetics-based epidemiological approach that allows for causal inference at the genetic level by replacing the corresponding traits with genetic variants [15,16]. It can compensate for the limitations of both observational studies and basic experiments.

Taken together, this MR study tried to use summary data of several common peripheral inflammatory factors (e.g. CRP, IL-1α, IL-1β, IL-6, IL-10 and IL-18) and inflammatory cells (e.g., neutrophil, lymphocyte, and monocyte counts) to explore the causal association of them with the risk of AMI. The study also planned to adopt summary data on AMI to explore the genetic effects of this disease on peripheral levels of inflammatory factors and cells.

## 2. Methods

### 2.1 Collection of summary data

The summary data for peripheral CRP was obtained from two genome-wide association studies (GWASs) (i.e., on HapMap and 1000 Genomes imputed data) of circulating amounts of CRP by using data from 88 studies comprising 204,402 European individuals [17]. The summary data for peripheral IL-1α and IL-1β were included in one study that characterized the genetic architecture of the human plasma proteome in 3,301 European healthy blood donors from the INTERVAL study, which identified 1,927 genetic associations with 1,478 proteins [18]. The data for peripheral IL-6 and IL-18 were obtained from a study that mapped and replicated protein quantitative traits for 90 cardiovascular proteins in 21,758 European individuals, resulting in 451 protein quantitative traits for 85 proteins [19]. The data for IL-10 was included in a GWAS that identified 27 loci influencing concentrations of circulating cytokines and growth factors in 7,681 European individuals [20]. The data for peripheral neutrophil, lymphocyte, and monocyte counts were obtained from a Blood Cell Consortium study using 563,946 European individuals.

The summary data for AMI was collected from a FinnGen study with 307,230 European individuals [21]. In this study, the cases were identified by the International Classification of Diseases (ICD) code on hospital or death records. The codes of ICD-10 were I21 and I22, while the codes of both ICD-9 and ICD-8 were 410.

### 2.2 Extraction of instrumental variables

Suitable instrumental variables, namely single nucleotide polymorphisms (SNPs), for each trait were derived from the corresponding GWAS mentioned above and satisfied the correlation, independence, and exclusivity assumptions [15,16]. Inclusion criteria were listed as follows: First, a genome-wide significance P-value for each SNP must be less than 5 × 10^–6^, along with a F-statistic greater than 10, thus satisfying the correlation assumption. Second, a SNP with linkage disequilibrium (a clumping window of 10 MB and an r^2^ cutoff of 0.001) was removed to satisfy the independence assumption. Third, a SNP related to the outcomes or potential confounders was also removed by the PhenoScanner V2 to satisfy the exclusivity assumption.

### 2.3 Data analysis

Two-sample MR analysis was performed using random-effect inverse-variance weighted (IVW), weighted median, and MR-Egger methods [15,16,22]. The IVW had the ability to report the most reliable results when horizontal pleiotropy was ignored. The weighted median and MR-Egger had a wider range of applicability, with the former allowing about 50% of instrumental variables to be horizontally pleiotropic and the latter tolerating all instrumental variables to be pleiotropic as long as these pleiotropies could not affect the correlation of these instrumental variables with the exposures. These two methods were therefore less precise than the IVW but can be used as a complement to the IVW. All three methods reported odds ratios (ORs), 95% confidence intervals (95%CIs) and P-values. A P-value less than 0.05 indicated that the correlation was nominally significant. Due to multiple comparisons of the ten inflammatory factors or cells, the Bonferroni correction was adopted. A Bonferroni-corrected P-value less than 0.005 (0.05/10) indicated that the correlation was statistically significant. In addition, forest plots and scatter plots were used to visualize the results.

Sensitivity analysis was performed using the following methods [23]. First, Cochran’s Q test was used to detect heterogeneity. Second, the MR-Egger intercept test was used to determine horizontal pleiotropy. Third, the leave-one-out method was used to assess the effect of each SNP on the pooled results, and any SNP with a disproportionate effect generally implied horizontal pleiotropy. Fourth, forest plots were used to graphically detect horizontal pleiotropy, and if their shape was relatively symmetrical, it meant that the instrumental variables were less affected by horizontal pleiotropy. Fifth, the MR-Pleiotropy Residual Sum and Outlier method (MR-PRESSO) was used to detect outliers, and removal of these outliers may correct horizontal pleiotropy. In addition, the MR-PRESSO can also make causal inferences and reported β values, 95%Cis, and P-values. A P-value less than 0.05 or 0.005 suggested that the correlation was nominally or statistically significant. It was noted that the MR-PRESSO results were more precise than the IVW results when the proportion of instrumental variables with horizontal pleiotropy was less than 10% of the total [24].

All data analyses were performed using R (version 1.4.1106) with the TwoSampleMR package (version 0.5.6).

## 3. Results

### 3.1 Overview of the study

As shown in Table 1, a series of SNPs were obtained to predict the traits in the study based on the three main assumptions of MR. Of these SNPs, 670 were used to predict lymphocyte count, while the number of SNPs predicting the other traits ranged from 9 to 670. In addition, the total F statistics for the SNPs were also listed in Table 1, and all of them were greater than 10.

**Table 1:** Data sources and characteristics for this study.^a^


TRAITS	YEAR	SOURCES	SAMPLE SIZE	POPULATION/SEX	NO. OF SUITABLE IVS	F STATISTICS

C-reactiveprotein	2018	PMID: 30388399Ligthart et al.	204,402	European/Both	110	41.771

Interleukin-1α	2018	PMID: 29875488Sun et al.	3,301	European/Both	10	10.251

Interleukin-1β	2018	PMID: 29875488Sun et al.	3,301	European/Both	9	10.461

Interleukin-6	2020	PMID: 33067605Folkersen et al.	21,758	European/Both	15	12.016

Interleukin-10	2016	PMID: 27989323Ahola-Olli et al.	7,681	European/Both	12	15.418

Interleukin-18	2020	PMID: 33067605Folkersen et al.	21,758	European/Both	24	20.058

Neutrophilcount	2020	BCCVuckovic et al.	563,946	European/Both	572	31.443

Lymphocytecount	2020	BCCVuckovic et al.	563,946	European/Both	670	32.688

Monocytecout	2020	BCCVuckovic et al.	563,946	European/Both	653	45.533

Acute myocardialinfarction	2022	FinnGenKurki et al.	307,230	European/Both	102	11.499


^a^ BCC = Blood Cell Consortium; IV = Instrumental variable.

### 3.2 Effect of inflammatory variables on the risk of AMI

In Table 2, the IVW and weighted median reported that the elevated peripheral level of IL-10 was nominally associated with a reduced risk of AMI (OR = 0.876, 95% CI = 0.788 ~ 0.974, P = 0.015; OR = 0.868, 95% CI = 0.767 ~ 0.981, P = 0.024), while the MR-Egger provided a non-significant result in the opposite direction (OR = 1.265, 95% CI = 0.572 ~ 2.798, P = 0.579). In Table 4, similar to the IVW and weighted median, the MR-PRESSO confirmed the nominal relationship between the increased level of IL-10 and the reduced risk of the disease (OR = 0.876, 95% CI = 0.788 ~ 0.974, P = 0.041).

**Table 2:** Effect of inflammatory factors and inflammatory cells on the risk of acute myocardial infarction.^a^


EXPOSURES	METHODS	OR (95%CI)	P VALUE^b^	P VALUE^c^	P VALUE^d^

C-reactiveprotein	IVW	0.972 (0.896 ~ 1.055)	0.504	0.027	0.298

	Weighted median	0.947 (0.842 ~ 1.066)	0.367		

	MR-Egger	0.920 (0.805 ~ 1.050)	0.220		

Interleukin-1α	IVW	1.008 (0.927 ~ 1.095)	0.857	0.301	0.121

	Weighted median	0.978 (0.885 ~ 1.080)	0.660		

	MR-Egger	0.353 (0.124 ~ 1.009)	0.124		

Interleukin-1β	IVW	0.996 (0.901 ~ 1.101)	0.938	0.092	0.226

	Weighted median	1.009 (0.910 ~ 1.119)	0.867		

	MR-Egger	1.359 (0.866 ~ 2.132)	0.239		

Interleukin-6	IVW	0.991 (0.888 ~ 1.106)	0.875	0.477	0.234

	Weighted median	0.954 (0.826 ~ 1.102)	0.522		

	MR-Egger	1.148 (0.895 ~ 1.473)	0.309		

Interleukin-10	IVW	**0.876 (0.788 ~ 0.974)**	**0.015**	0.171	0.390

	Weighted median	**0.868 (0.767 ~ 0.981)**	**0.024**		

	MR-Egger	1.265 (0.572 ~ 2.798)	0.579		

Interleukin-18	IVW	**0.934 (0.875 ~ 0.997)**	**0.040**	0.603	0.503

	Weighted median	**0.916 (0.840 ~ 0.998)**	**0.045**		

	MR-Egger	0.898 (0.787 ~ 1.024)	0.125		

Neutrophilcount	IVW	1.022 (0.952 ~ 1.097)	0.549	0.063	0.352

	Weighted median	1.053 (0.950 ~ 1.167)	0.325		

	MR-Egger	1.099 (0.928 ~ 1.303)	0.273		

Lymphocytecount	IVW	1.060 (0.992 ~ 1.131)	0.084	0.072	0.731

	Weighted median	1.029 (0.930 ~ 1.139)	0.580		

	MR-Egger	1.084 (0.936 ~ 1.255)	0.280		

Monocytecout	IVW	1.047 (0.992 ~ 1.106)	0.095	0.063	0.062

	Weighted median	1.023 (0.929 ~ 1.127)	0.643		

	MR-Egger	0.969 (0.879 ~ 1.069)	0.531		


^a^ IVW = Inverse variance weighted; OR = Odds ratio; CI = Confidence interval. ^b^ P value for Mendelian randomization. ^c^ P value for Cochran’s Q test. ^d^ P value for MR-Egger intercept test.

In Table 2, the IVW and weighted median, but not MR-Egger, reported that the elevated peripheral level of IL-18 was nominally related to a decreased risk of AMI (OR = 0.934, 95% CI = 0.875 ~ 0.997, P = 0.040; OR = 0.916, 95% CI = 0.840 ~ 0.998, P = 0.045; OR = 0.898, 95% CI = 0.787 ~ 1.024, P = 0.125). In Table 4, the MR-PRESSO supported the results from the IVW and weighted median (OR = 0.934, 95% CI = 0.879 ~ 0.993, P = 0.042).

**Figure 1:**
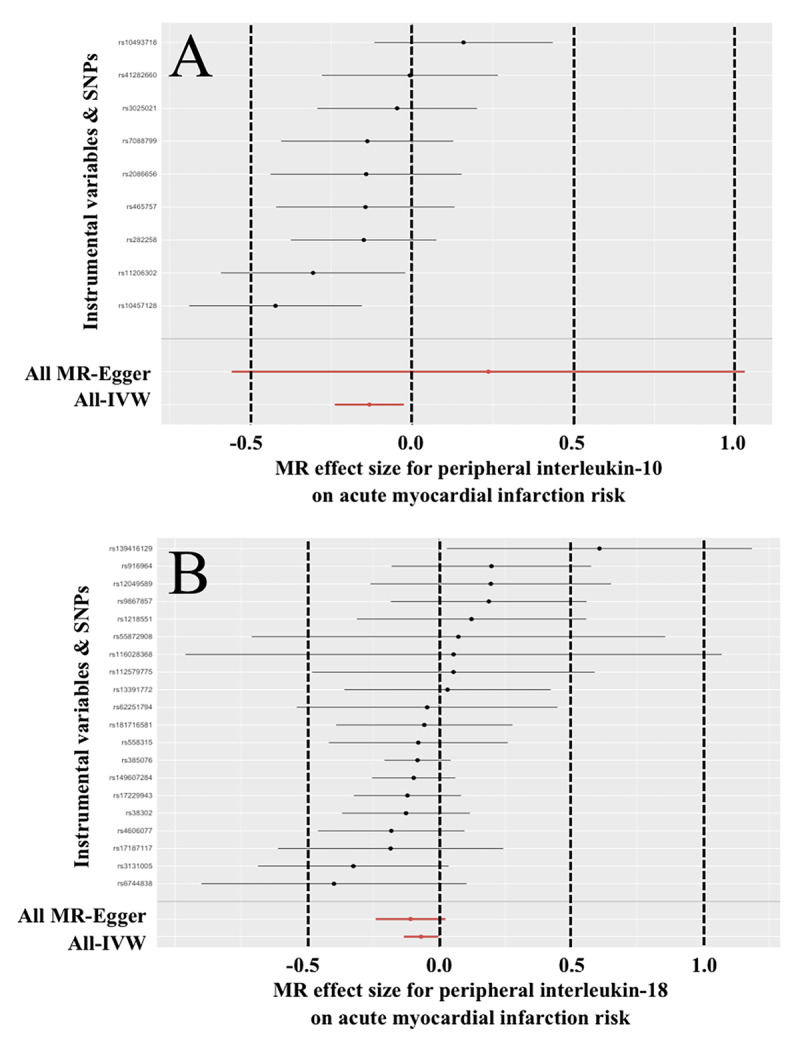
**Effect of peripheral interleukin-10 and interleukin 18 on the risk of acute myocardial infarction.** IVW = Inverse variance weighted; MR = Mendelian randomization; SNP = Single nucleotide polymorphism.

The results for IL-10 and IL-18 were visualized in Figure 1. In the sensitivity analyses for these two inflammatory factors, the MR-PRESSO removed significant outliers (Table 4). Then, the Cochran’s Q test did not find significant heterogeneity, and the MR-Egger intercept test, leave-one-out method, and forest plots did not detect significant horizontal pleiotropy (Table 2, Supplemental Figure 5, and Supplemental Figure 6).

In Table 2, no significant correlation was found between other inflammatory variables and the risk of AMI in this study (P > 0.05).

### 3.3 Effect of AMI on periphery levels of inflammatory variables

In Tables 3 and 4, the IVW and MR-PRESSO reported that the risk of AMI nominally increased the peripheral level of IL-10 (OR = 1.062, 95% CI = 1.003 ~ 1.124, P = 0.040; OR = 1.062, 95% CI = 1.003 ~ 1.124, P = 0.044), while the weighted median and MR-Egger did not support these findings (OR = 1.008, 95% CI = 0.925 ~ 1.099, P = 0.848; OR = 1.058, 95% CI = 0.917 ~ 1.221, P = 0.440).

**Table 3:** Effect of acute myocardial infarction on periphery levels of inflammatory factors and inflammatory cells.^a^


OUTCOMES	METHODS	OR (95%CI)	P VALUE^b^	P VALUE^c^	P VALUE^d^

C-reactiveprotein	IVW	1.004 (0.982 ~ 1.026)	0.739	0.146	0.097

	Weighted median	0.976 (0.949 ~ 1.004)	0.093		

	MR-Egger	0.955 (0.898 ~ 1.015)	0.147		

Interleukin-1α	IVW	1.009 (0.927 ~ 1.097)	0.844	0.118	0.782

	Weighted median	0.931 (0.824 ~ 1.051)	0.248		

	MR-Egger	1.037 (0.837 ~ 1.285)	0.740		

Interleukin-1β	IVW	0.941 (0.870 ~ 1.017)	0.122	0.553	0.665

	Weighted median	0.888 (0.789 ~ 1.001)	0.052		

	MR-Egger	0.979 (0.805 ~ 1.191)	0.833		

Interleukin-6	IVW	0.992 (0.954 ~ 1.031)	0.671	0.500	0.401

	Weighted median	0.996 (0.934 ~ 1.062)	0.906		

	MR-Egger	0.954 (0.865 ~ 1.052)	0.348		

Interleukin-10	IVW	**1.062 (1.003 ~ 1.124)**	**0.040**	0.404	0.961

	Weighted median	1.008 (0.925 ~ 1.099)	0.848		

	MR-Egger	1.058 (0.917 ~ 1.221)	0.440		

Interleukin-18	IVW	0.979 (0.943 ~ 1.015)	0.250	0.058	0.148

	Weighted median	0.961 (0.913 ~ 1.012)	0.134		

	MR-Egger	0.919 (0.838 ~ 1.008)	0.075		

Neutrophilcount	IVW	0.995 (0.987 ~ 1.003)	0.244	0.054	0.252

	Weighted median	0.989 (0.978 ~ 1.002)	0.088		

	MR-Egger	0.985 (0.967 ~ 1.004)	0.127		

Lymphocytecount	IVW	0.993 (0.985 ~ 1.001)	0.083	0.153	0.455

	Weighted median	**0.982 (0.970 ~ 0.994)**	**0.003**		

	MR-Egger	0.987 (0.969 ~ 1.005)	0.155		

Monocytecout	IVW	1.000 (0.991 ~ 1.009)	0.995	0.149	0.835

	Weighted median	0.994 (0.981 ~ 1.007)	0.356		

	MR-Egger	0.998 (0.978 ~ 1.019)	0.853		


^a^ IVW = Inverse variance weighted; OR = Odds ratio; CI = Confidence interval. ^b^ P value for Mendelian randomization. ^c^ P value for Cochran’s Q test. ^d^ P value for MR-Egger intercept test.

**Table 4:** MR-PRESSO results for Mendelian randomization analyses and outlier tests.^a^


EXPOSURES	β VALUE	SD	OR (95%CI)	P VALUE FOR MR	P VALUE FOR OUTLIERS

C-reactive protein	–0.028	0.042	0.972 (0.896 ~ 1.056)	0.506	0.032

Interleukin-1α	0.008	0.042	1.008 (0.928 ~ 1.095)	0.864	0.342

Interleukin-1β	–0.004	0.051	0.996 (0.901 ~ 1.101)	0.941	0.124

Interleukin-6	–0.009	0.055	0.991 (0.890 ~ 1.104)	0.875	0.498

Interleukin-10	**–0.132**	**0.054**	**0.876 (0.788 ~ 0.974)**	**0.041**	0.229

Interleukin-18	**–0.068**	**0.031**	**0.934 (0.879 ~ 0.993)**	**0.042**	0.665

Neutrophil count	0.022	0.036	1.022 (0.953 ~ 1.097)	0.550	0.064

Lymphocyte count	0.058	0.033	1.060 (0.993 ~ 1.131)	0.084	0.076

Monocyte cout	0.046	0.028	1.047 (0.991 ~ 1.106)	0.096	0.070

**OUTCOMES**	**β VALUE**	**SD**	**OR (95%CI)**	**P VALUE FOR MR**	**P VALUE FOR OUTLIERS**

C-reactive protein	0.004	0.011	1.004 (0.983 ~ 1.026)	0.741	0.145

Interleukin-1α	0.008	0.043	1.008 (0.927 ~ 1.097)	0.844	0.116

Interleukin-1β	–0.061	0.039	0.941 (0.872 ~ 1.016)	0.121	0.567

Interleukin-6	–0.008	0.020	0.992 (0.954 ~ 1.032)	0.671	0.500

Interleukin-10	**0.060**	**0.029**	**1.062 (1.003 ~ 1.124)**	**0.044**	0.418

Interleukin-18	–0.022	0.019	0.978 (0.942 ~ 1.015)	0.253	0.057

Neutrophil count	–0.005	0.004	0.995 (0.987 ~ 1.003)	0.249	0.053

Lymphocyte count	–0.007	0.004	0.993 (0.985 ~ 1.001)	0.089	0.138

Monocyte cout	2.733e-05	0.004	1.000 (0.992 ~ 1.008)	0.995	0.138


^a^ SD = Standard deviation; OR = Odds ratio; CI = Confidence interval; MR = Mendelian randomization.

**Figure 2:**
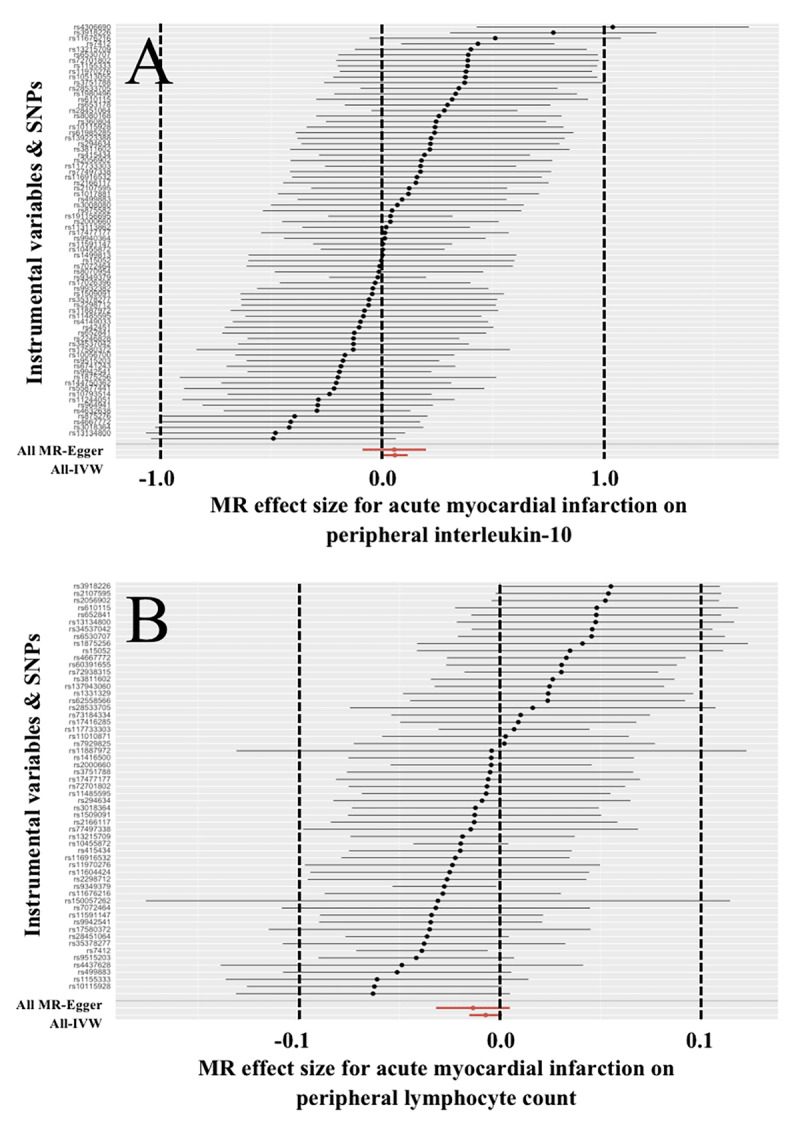
**Effect of acute myocardial infarction on peripheral interleukin-10 and lymphocyte count.** IVW = Inverse variance weighted; MR = Mendelian randomization; SNP = Single nucleotide polymorphism.

The results for IL-10 were visualized in Figure 2. In the sensitivity analyses for IL-10, the MR-PRESSO removed significant outliers (Table 4). Then, the Cochran’s Q test reported no significant heterogeneity, and the MR-Egger intercept test, leave-one-out method, and forest plots detected no significant horizontal pleiotropy (Table 3, Supplemental Figure 14).

In Table 3, the weighted median reported that the risk of AMI significantly decreased the peripheral level of lymphocyte count (OR = 0.982, 95% CI = 0.970 ~ 0.994, P = 0.003). However, the IVW, MR-Egger, and MR-PRESSO did not reported any similar results (OR = 0.993, 95% CI = 0.985 ~ 1.001, P = 0.083; OR = 0.987, 95% CI = 0.969 ~ 1.005, P = 0.155; OR = 0.993, 95%CI = 0.985 ~ 1.001, P = 0.089). These results were visualized in Figure 2.

In Table 3, the risk of AMI did not affect the peripheral levels of any other inflammatory variables (P > 0.05).

## 4. Discussion

At present, the mortality rate of AMI has been gradually decreasing [25]. However, the health risk associated with the disease is still enormous. In the absence of timely treatment, AMI remains a highly lethal cardiovascular disease. Thus, understanding its pathogenesis and pathophysiological processes is necessary to effectively prevent the disease and improve its prognosis, and inflammation is one of the unavoidable topics. To our knowledge, the present study was the first published MR study worldwide to explore the bidirectional association of AMI with several common peripheral inflammatory factors and inflammatory cells, and its findings may contribute to a better understanding of the relationship between inflammation and AMI, which is of great scientific significance.

With the exception of the MR-Egger, which had the worst precision, several methods (including the IVW) reported an approximately 10% to 15% decreased risk of AMI for each SD increase in peripheral levels of IL-10 and IL-18. And two methods with better precision (i.e., the IVW and MR-PRESSO) also reported that the risk of AMI increased the peripheral IL-10 level by approximately 6%, although the other two methods did not support these results. Because the sensitivity analyses had excluded the significant effect of horizontal pleiotropy, we considered the results of the IVW and MR-PRESSO to be more reliable and endorsed them as the final results of the study. In addition, the weighted median reported that the risk of AMI reduced the peripheral lymphocyte count level, but other methods such as the IVW did not yield similar results. Therefore, this study considered that there was no sufficient evidence to justify this correlation.

It is well known that IL-10 is an important inflammatory suppressor in the body and is implicated in the development and progression of cardiovascular diseases. Several basic experiments demonstrated that IL-10 can promote myocardial repair, inhibit cardiac remodeling, and ameliorate functional impairment of other important organs after AMI [26,27,28]. Based on the findings from the present study and previous experiments, we suggested that IL-10 was likely to prevent the development of AMI; following the disease, the inflammatory factor might in turn be upregulated in a feedback manner, serving to promote repair and improve prognosis.

IL-18 belongs to the IL-1 family and has the ability to stimulate T cell proliferation, enhance natural killer cell activity, and contribute to the development of inflammatory responses. Many studies reported that IL-18 can promote the occurrence and worsening of cardiovascular events, and some scholars even regarded it as a potential therapeutic target for AMI [29,30]. However, several other studies provided different results. Kariž et al. suggested that IL-18 promoter gene polymorphisms were not risk factors for AMI in Caucasians [31]. Gao et al. reported that peripheral IL-18 levels on admission may not predict adverse events in patients with AMI undergoing percutaneous coronary intervention [32]. Thus, the role of IL-18 in AMI was complex and remained inconclusive. Moreover, results at the phenotypic level were susceptible to multiple factors and may be inconsistent with those at the genetic level. Therefore, more studies exploring the association of this inflammatory factor with AMI and the mechanisms involved were necessary.

The main advantage of this study was the MR approach. It completely avoided the reverse causality and effectively prevented the interference of most confounding factors. Thus, the results from this study were more reliable than those from previous observational studies. Second, this study was not isolated research but an updated study based on the previous observational studies. For example, all the inflammatory factors and cells included in the study were confirmed at the phenotypic level to be related to AMI by previous observational studies, and some of the results from this study may also be corroborated by previous observational studies. Third, the design of this study can also find some examples of concrete implementations in previous MR studies. For example, one previous MR study explored the interaction of 41 systemic inflammatory regulators with Alzheimer’s disease and showed that specific regulators may be downstream effects of this cognitive disease [33]. Another published MR study focused on the association of 47 inflammatory cytokines with five cancers, adding to the current knowledge of the role of specific inflammatory biomarker pathways in cancer etiology [34]. Therefore, this design was effective and can help us achieve the purpose of the study.

There were several limitations to the study. First, because the number of instrumental variables for some traits was not enough, the genome-wide correlation strength was related to 5 × 10^–6^. Meanwhile, the F statistic was adopted to exclude weak instrumental variables. These measures not only allowed us to obtain sufficient instrumental variables but also ensured that the correlation assumptions of MR were met. Therefore, we did not believe that this limitation affected the reliability of this study. Second, due to the lack of summary data, some important inflammatory factors, such as tumor necrosis factor-α, were not included in this study. Because these factors were likely to be associated with AMI, the correlation of inflammation with AMI might be underestimated in the study. Third, the study focused on the peripheral levels of the inflammatory factors, but the peripheral levels and myocardial tissue levels of the same factor may be different. Therefore, it was necessary to explore the relationship between the levels of inflammatory factors in myocardial tissue and AMI.

In conclusion, this study confirmed that both IL-10 and IL-18 were peripheral inflammatory factors genetically associated with AMI. In particular, combined with previous knowledge, IL-10 may have a protective effect on the onset, progression, and prognosis of the disease. Though this study also reported that IL-18 might have a protective effect on the onset of AMI, this was inconsistent with the phenotypic results from previous observational studies. In addition, this study obtained some interesting, but not sufficient, evidence to prove that the risk of AMI might genetically reduce peripheral lymphocyte count. All these findings should be validated by future studies.

## Data Accessibility Statement

This is a Mendelian randomisation study using public data. Data sources have been provided in the article and supplemental materials.

## Additional File

The additional file for this article can be found as follows:

10.5334/gh.1269.s1 Supplementary Files. Supplemental Figures 1 to 18.
