# Supplementary material for: Peripheral Inflammatory Factors and Acute Myocardial Infarction Risk: A Mendelian Randomization Study
Source: Glob Heart. 2023 Oct 6;18(1):55. doi: 10.5334/gh.1269 (PMC10558024; doi:10.5334/gh.1269)
Supplement: Supplementary Files. — Supplemental Figures 1 to 18. [file gh-18-1-1269-s1.pdf]

Supplementary Files

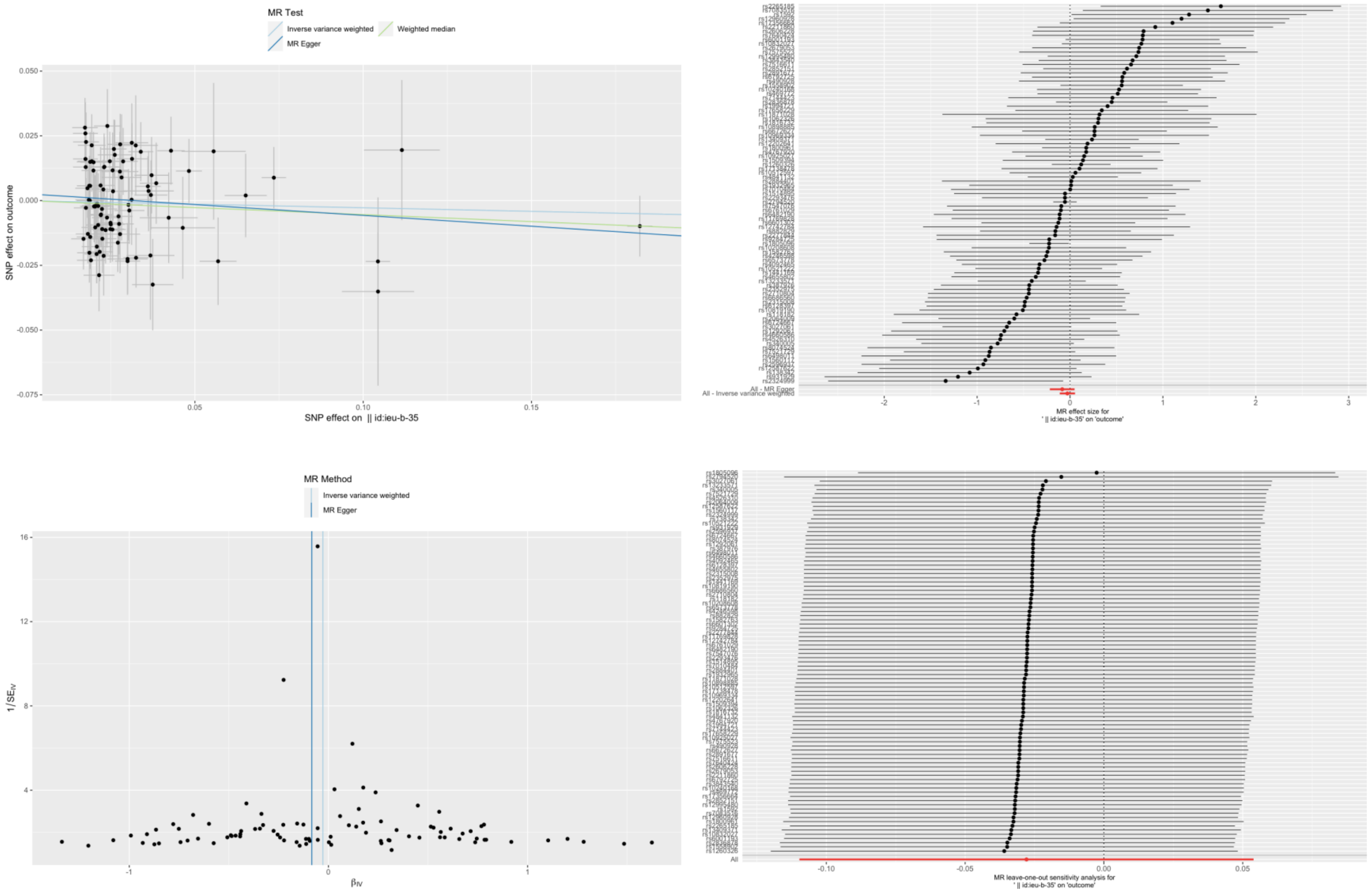

Supplemental Figure 1 Effect of peripheral C-reactive protein on myocardial infarction risk

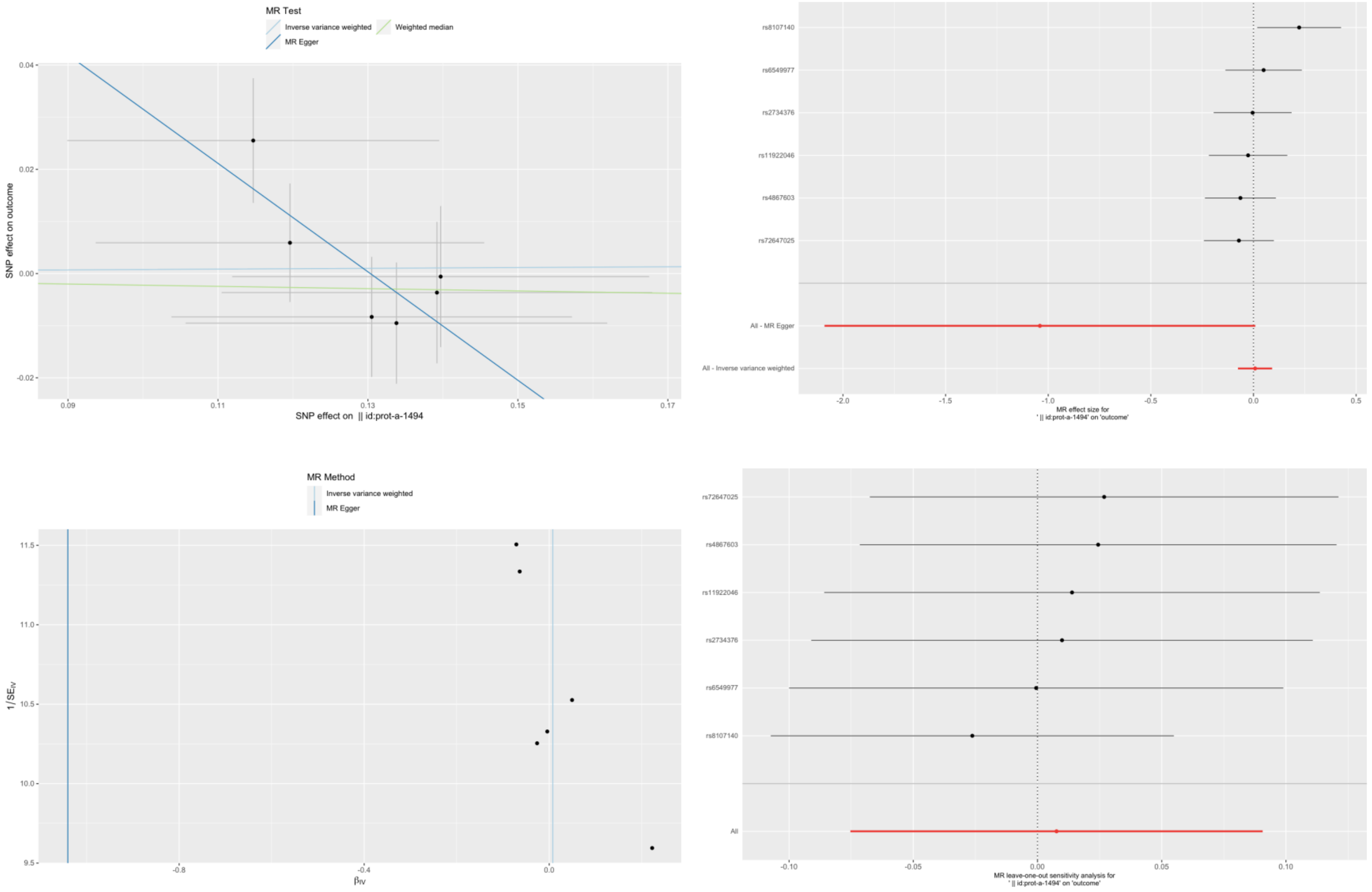

Supplemental Figure 2 Effect of peripheral interleukin-1α on myocardial infarction risk

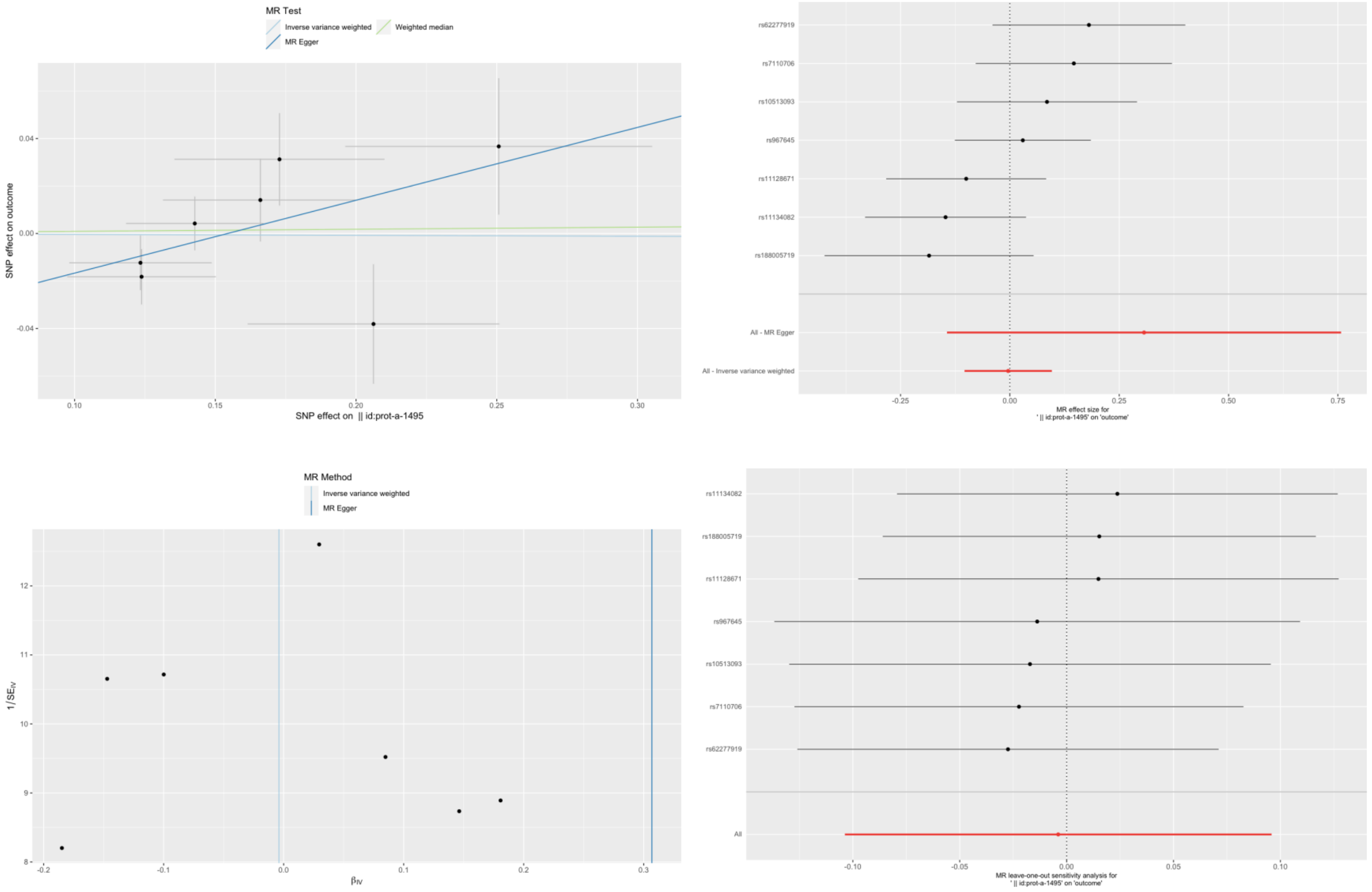

Supplemental Figure 3 Effect of peripheral interleukin-1β on myocardial infarction risk

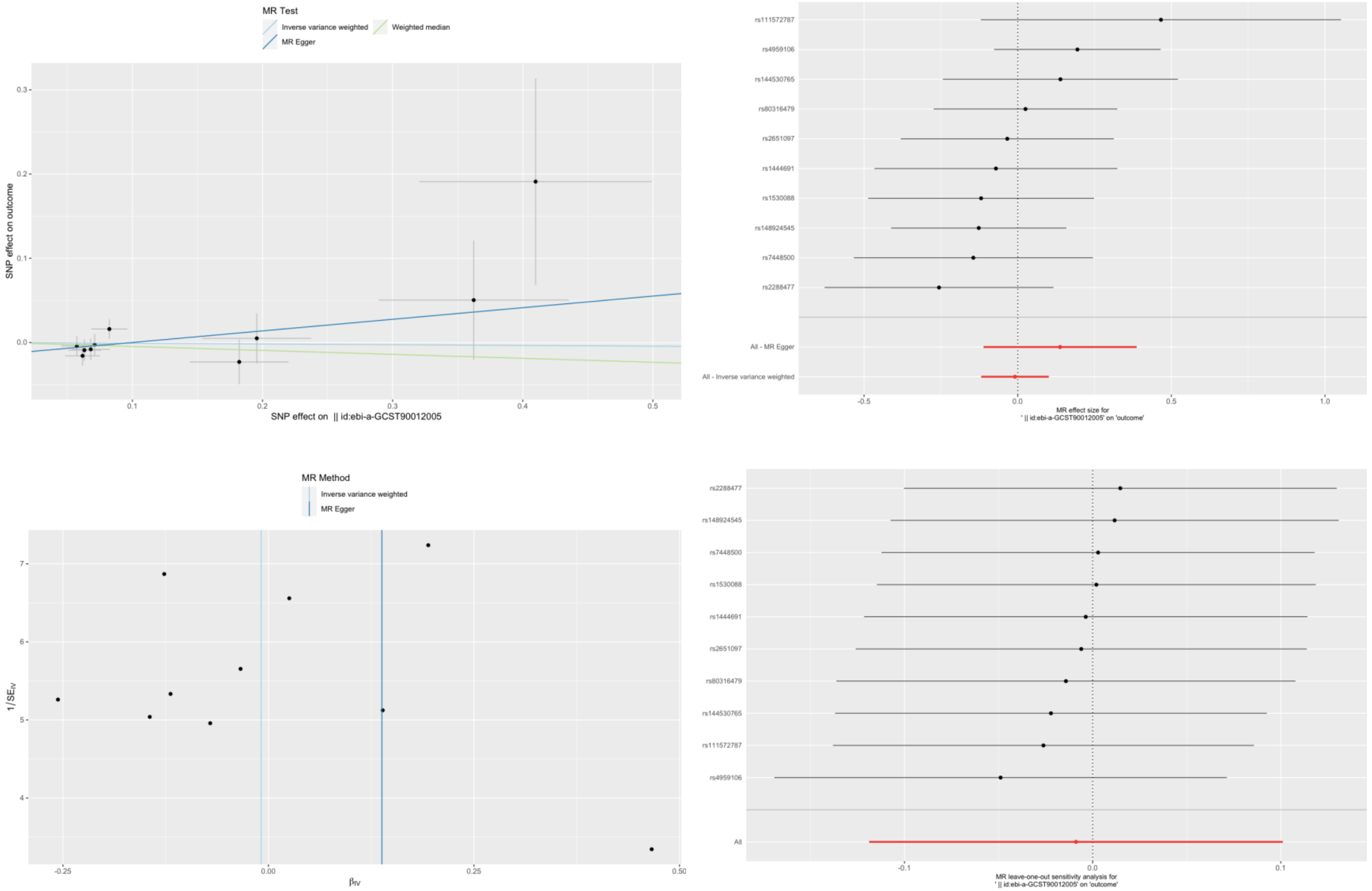

Supplemental Figure 4 Effect of peripheral interleukin-6 on myocardial infarction risk

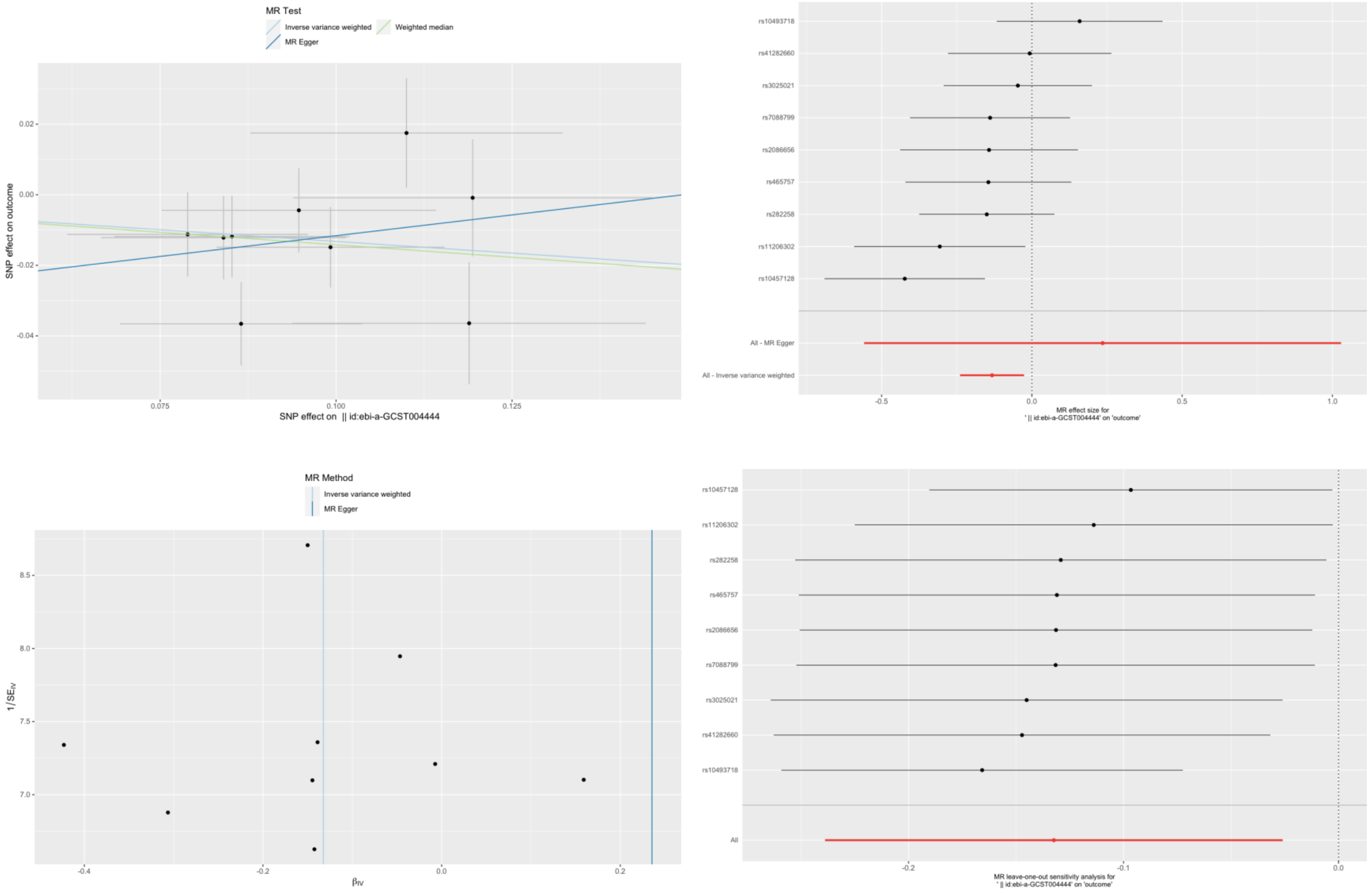

Supplemental Figure 5 Effect of peripheral interleukin-10 on myocardial infarction risk

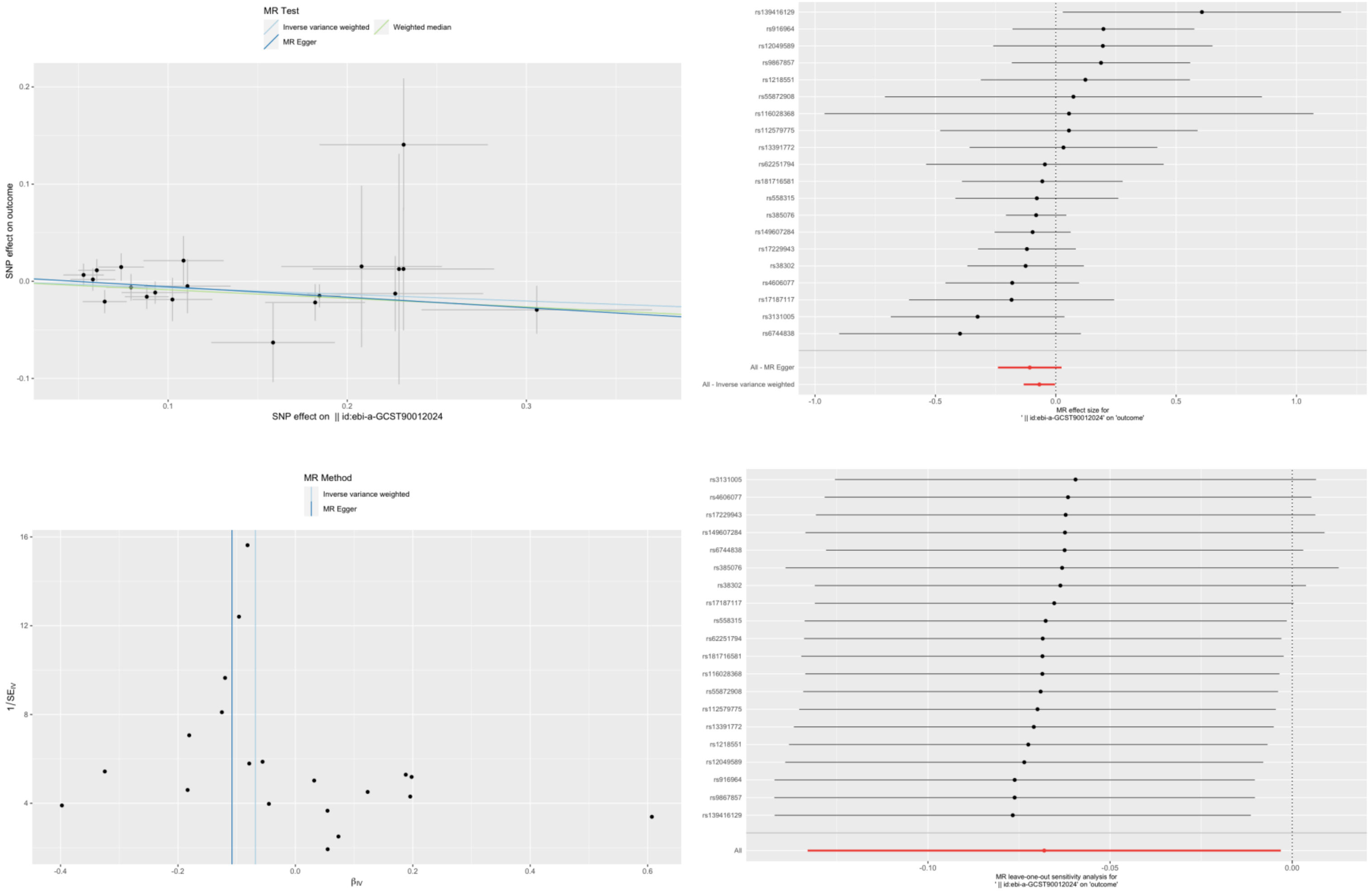

Supplemental Figure 6 Effect of peripheral interleukin-18 on myocardial infarction risk

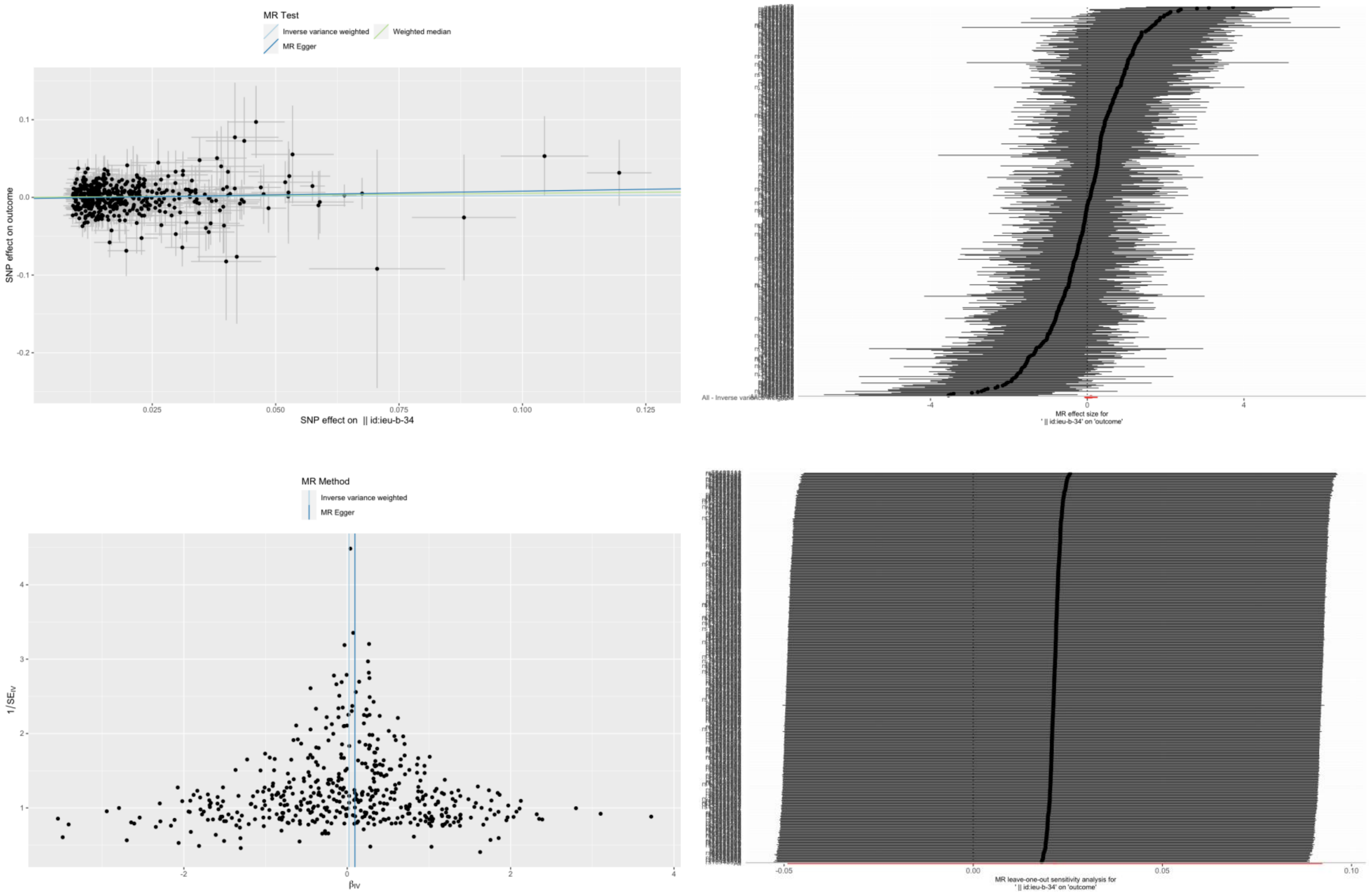

Supplemental Figure 7 Effect of peripheral neutrophil count on myocardial infarction risk

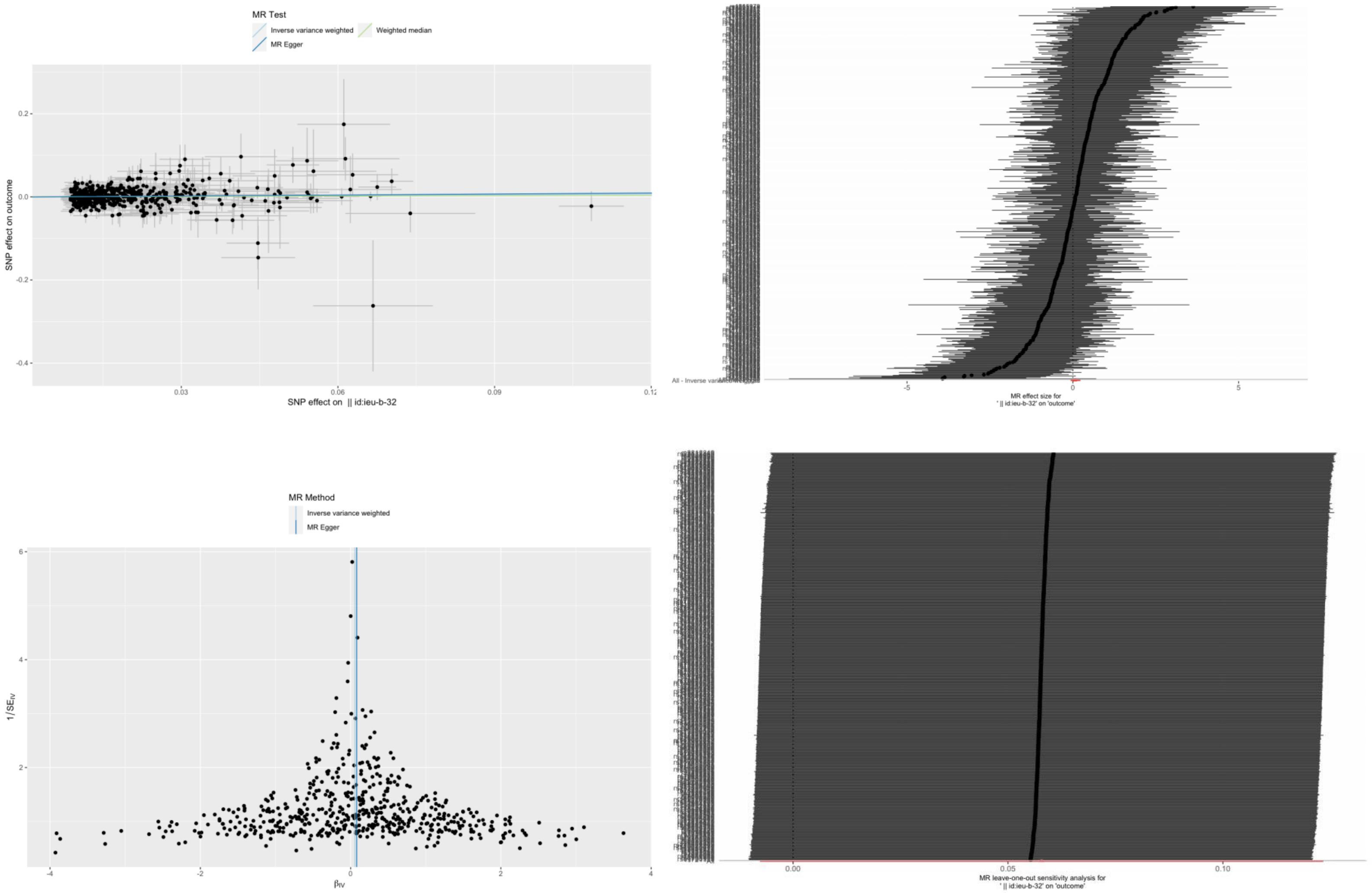

Supplemental Figure 8 Effect of peripheral lymphocyte count on myocardial infarction risk

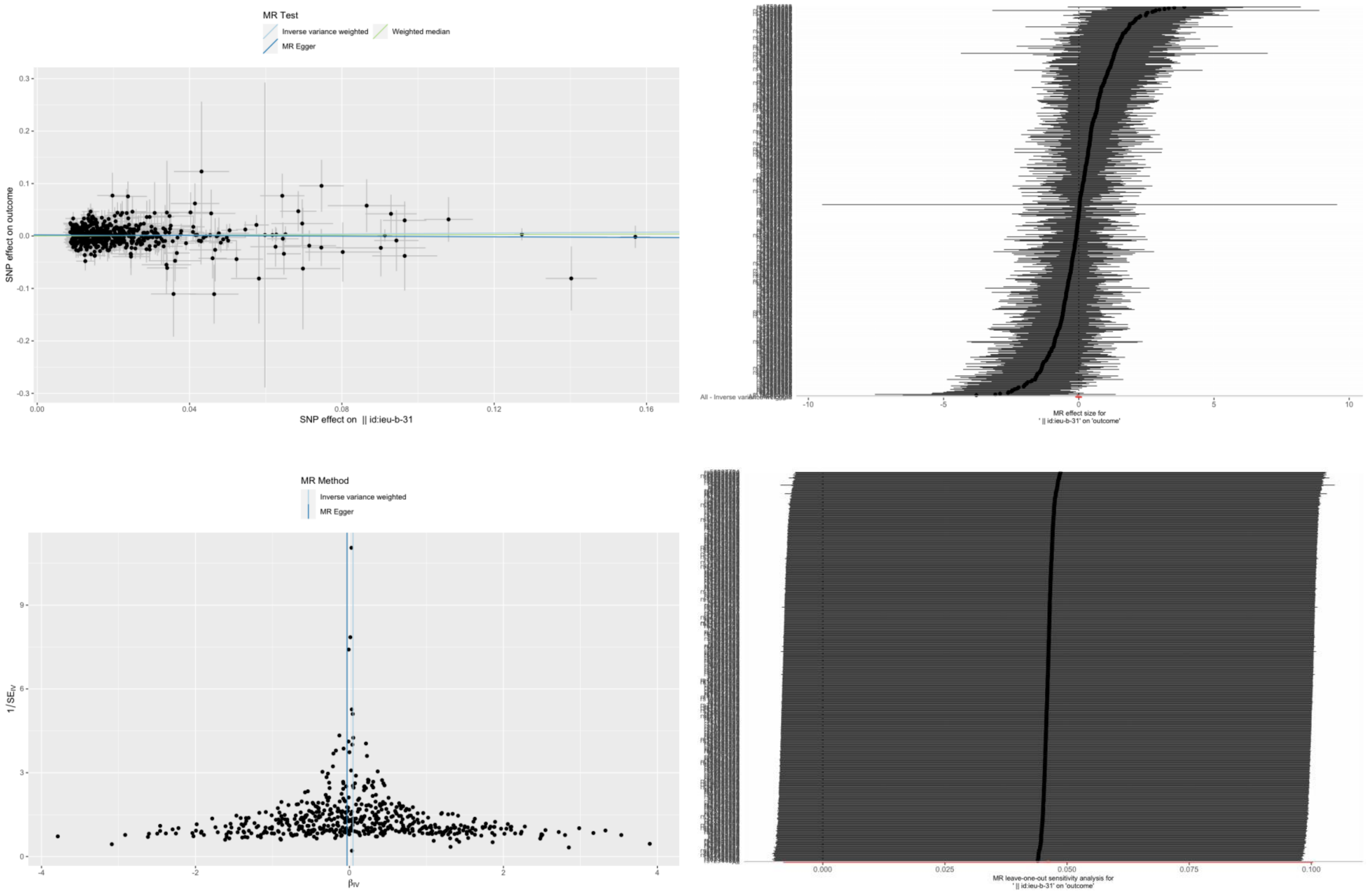

Supplemental Figure 9 Effect of peripheral monocyte count on myocardial infarction risk

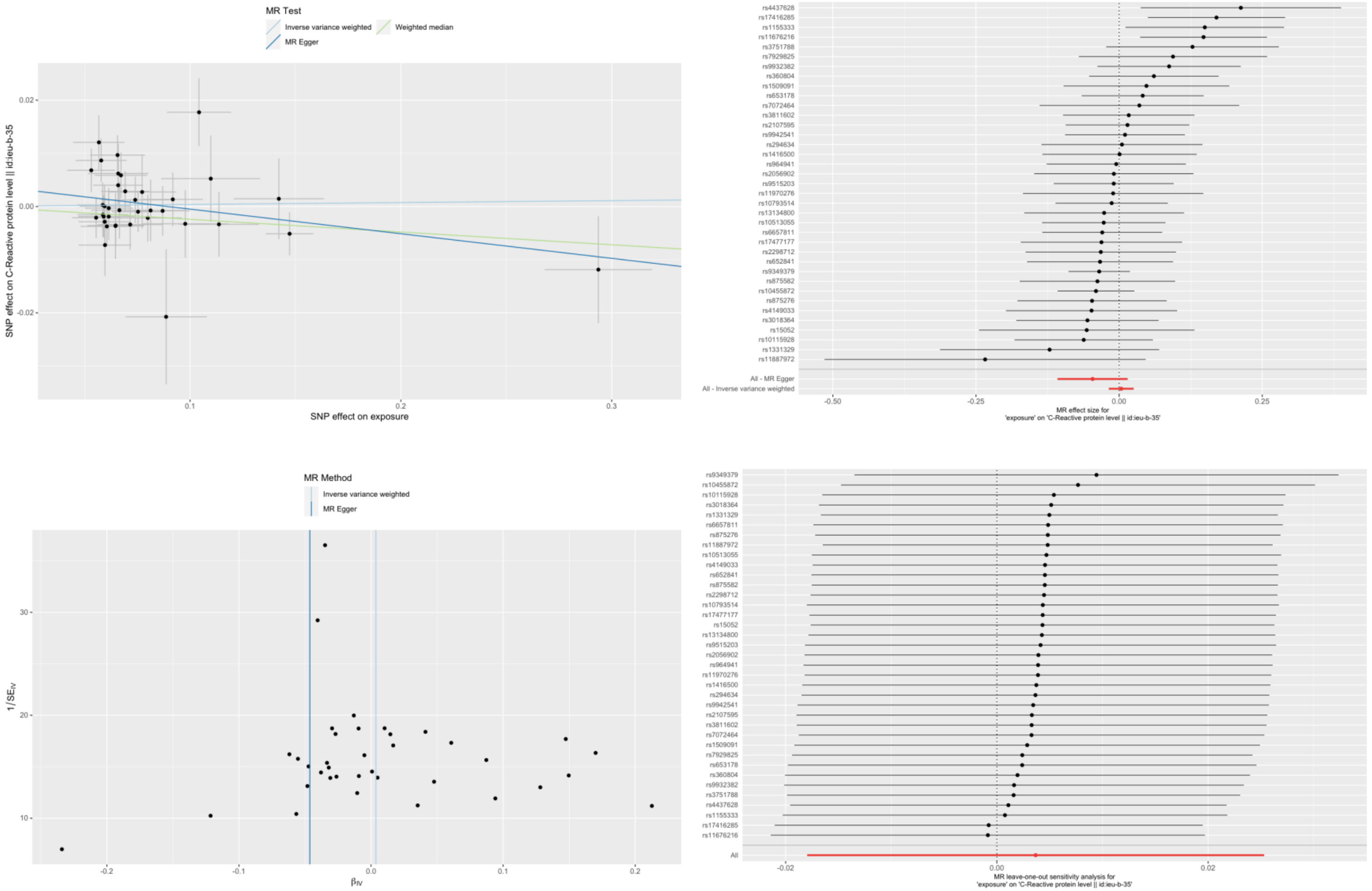

Supplemental Figure 10 Effect of myocardial infarction on peripheral C-reactive protein

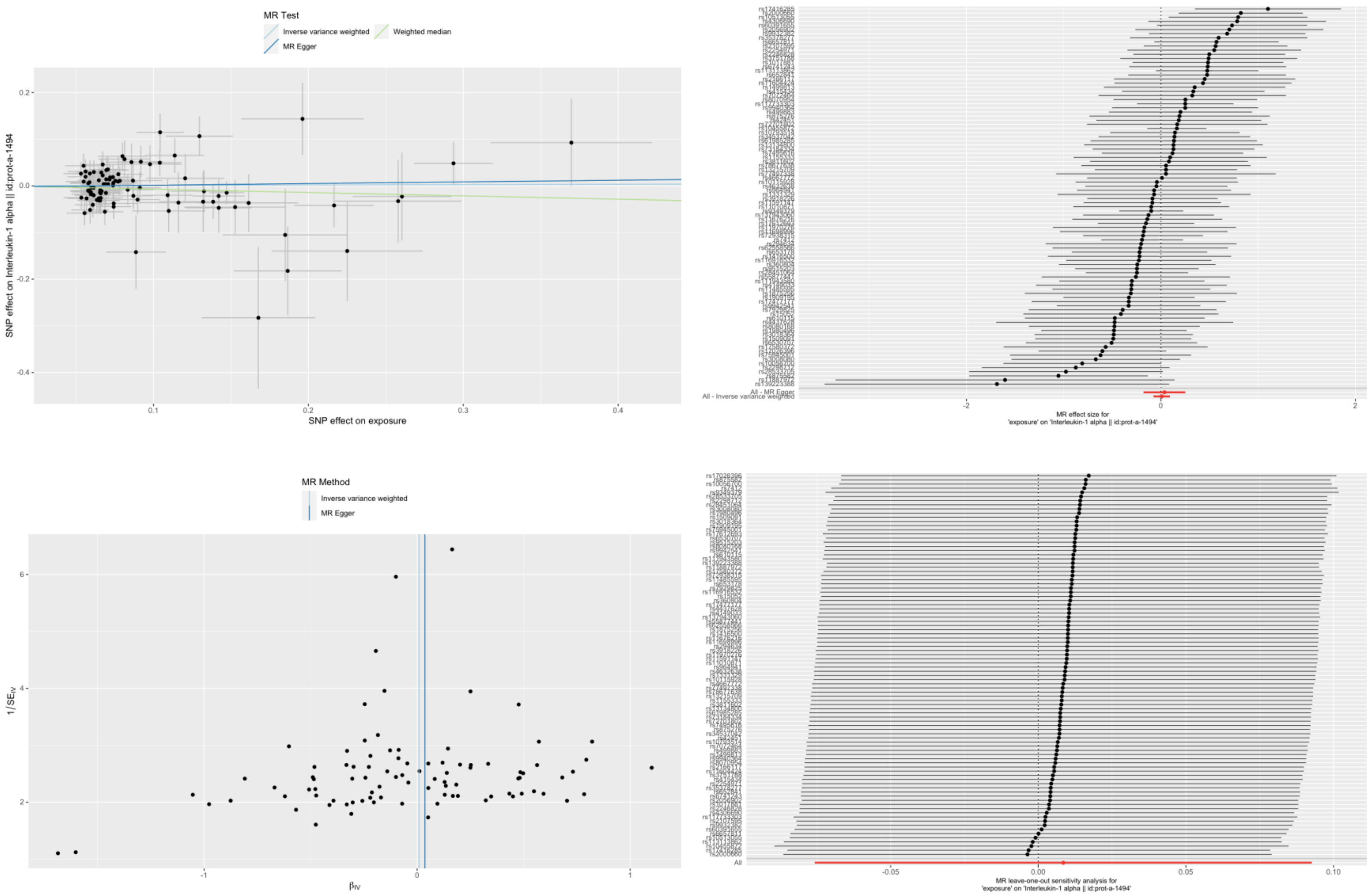

Supplemental Figure 11 Effect of myocardial infarction on peripheral interleukin-1α

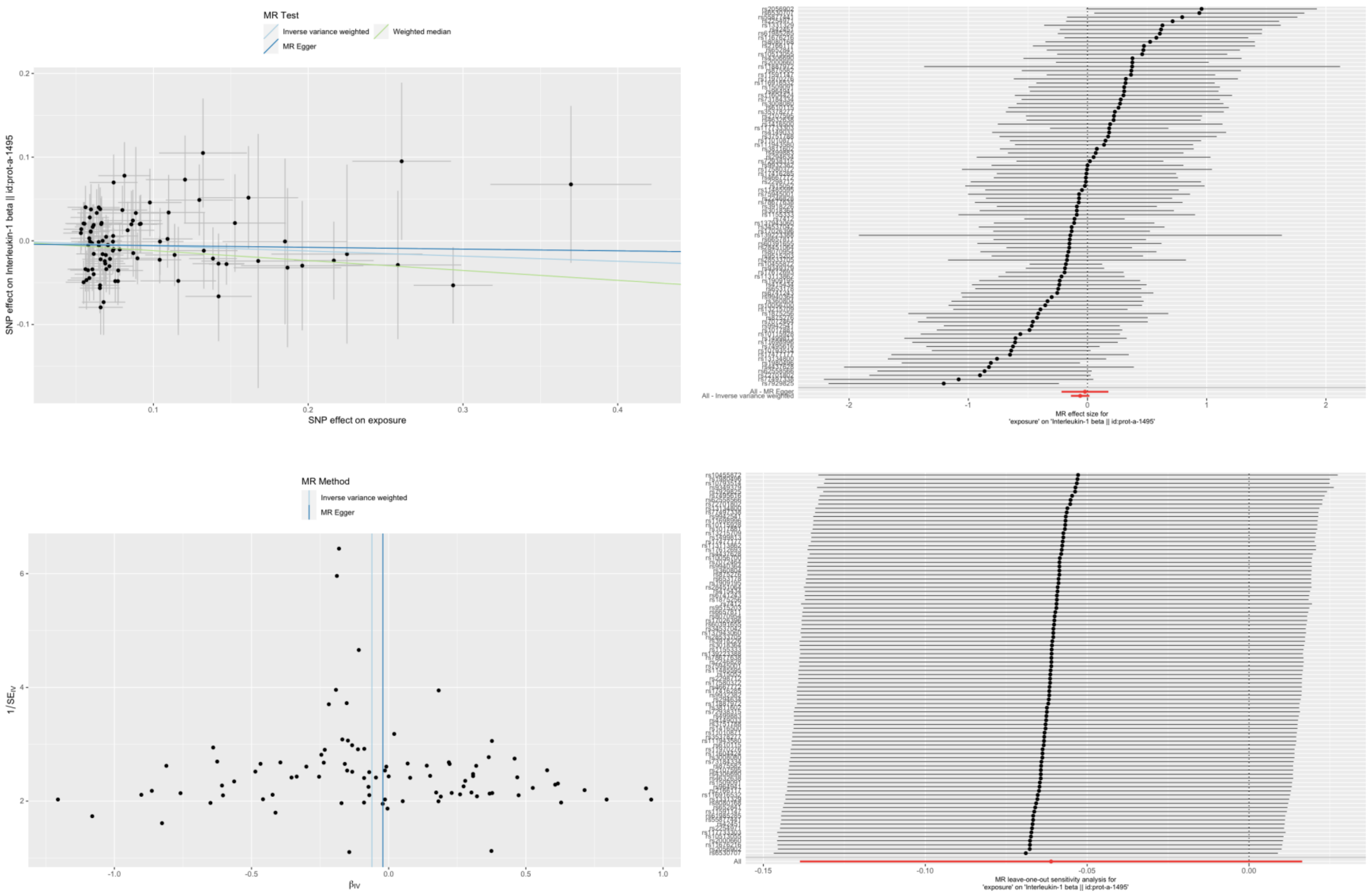

Supplemental Figure 12 Effect of myocardial infarction on peripheral interleukin-1β

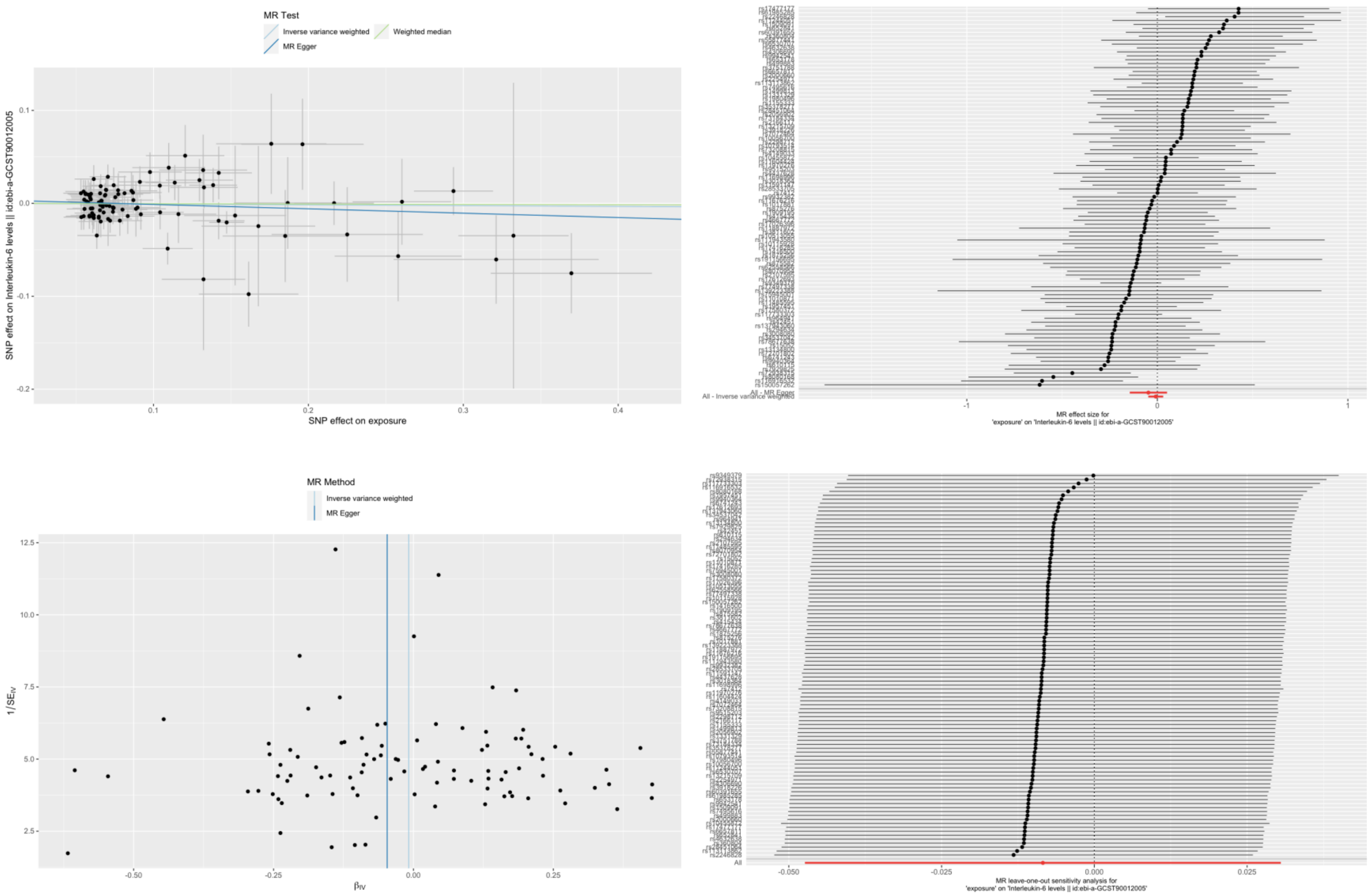

Supplemental Figure 13 Effect of myocardial infarction on peripheral interleukin-6

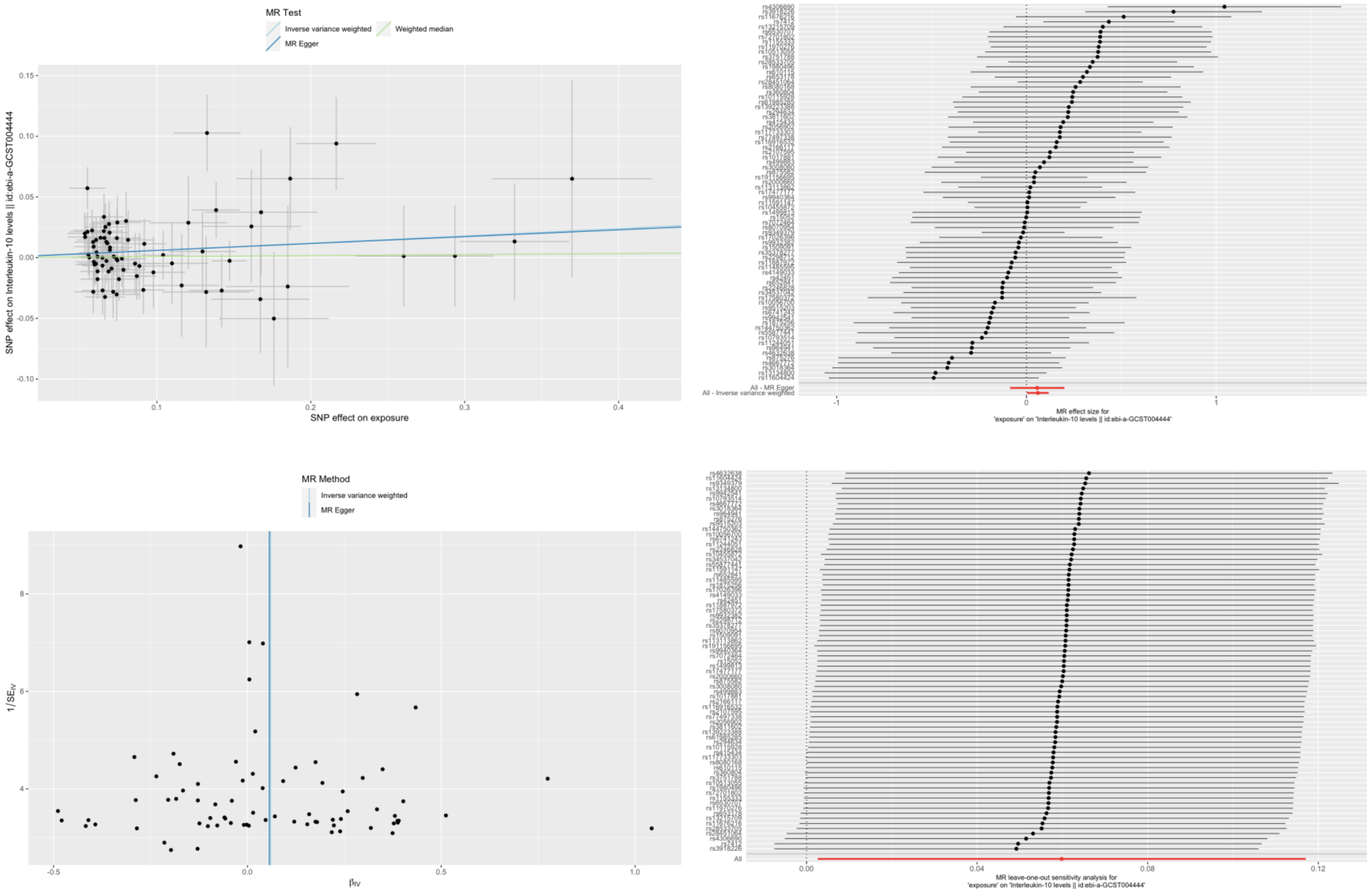

Supplemental Figure 14 Effect of myocardial infarction on peripheral interleukin-10

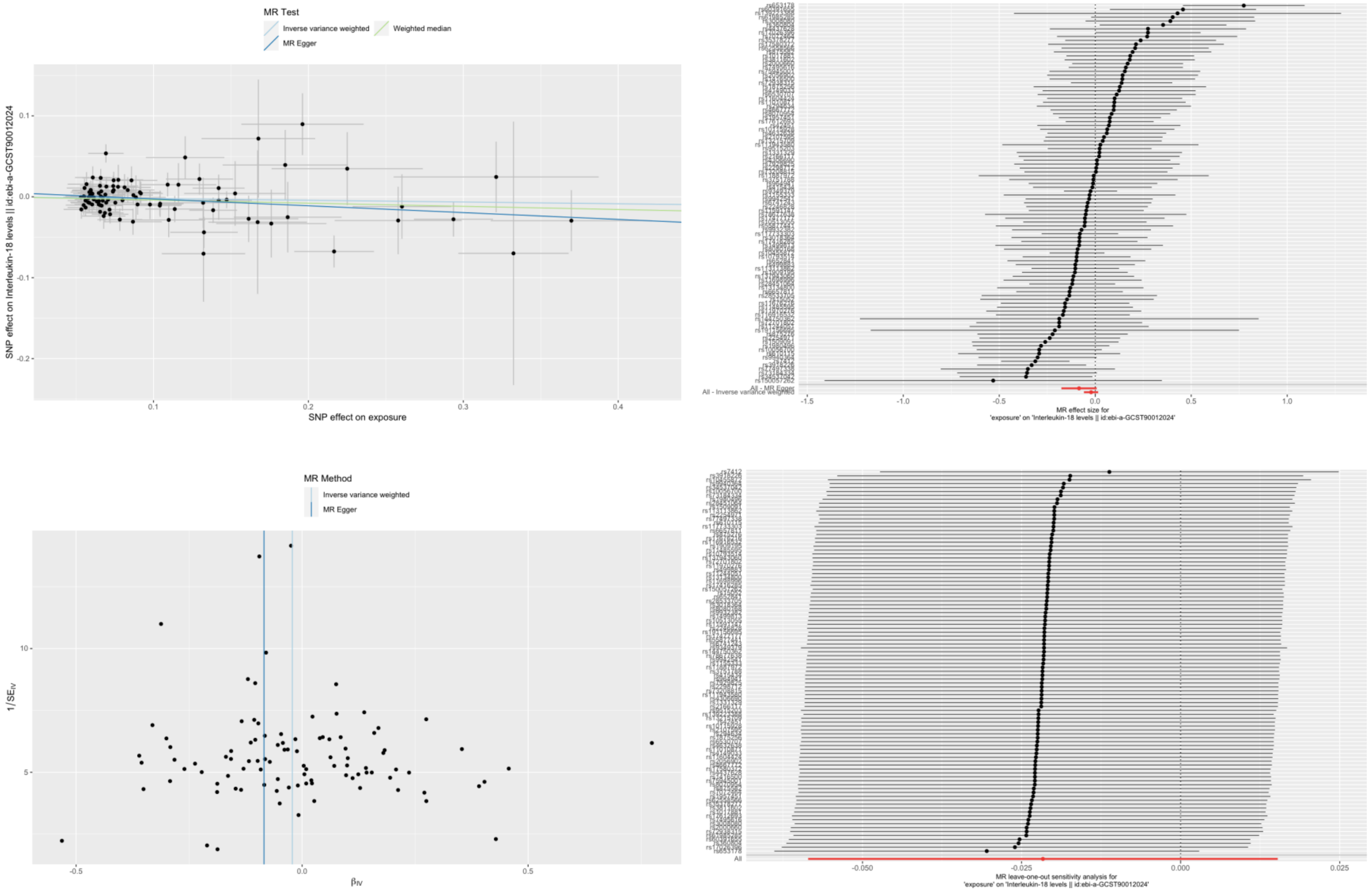

Supplemental Figure 15 Effect of myocardial infarction on peripheral interleukin-18

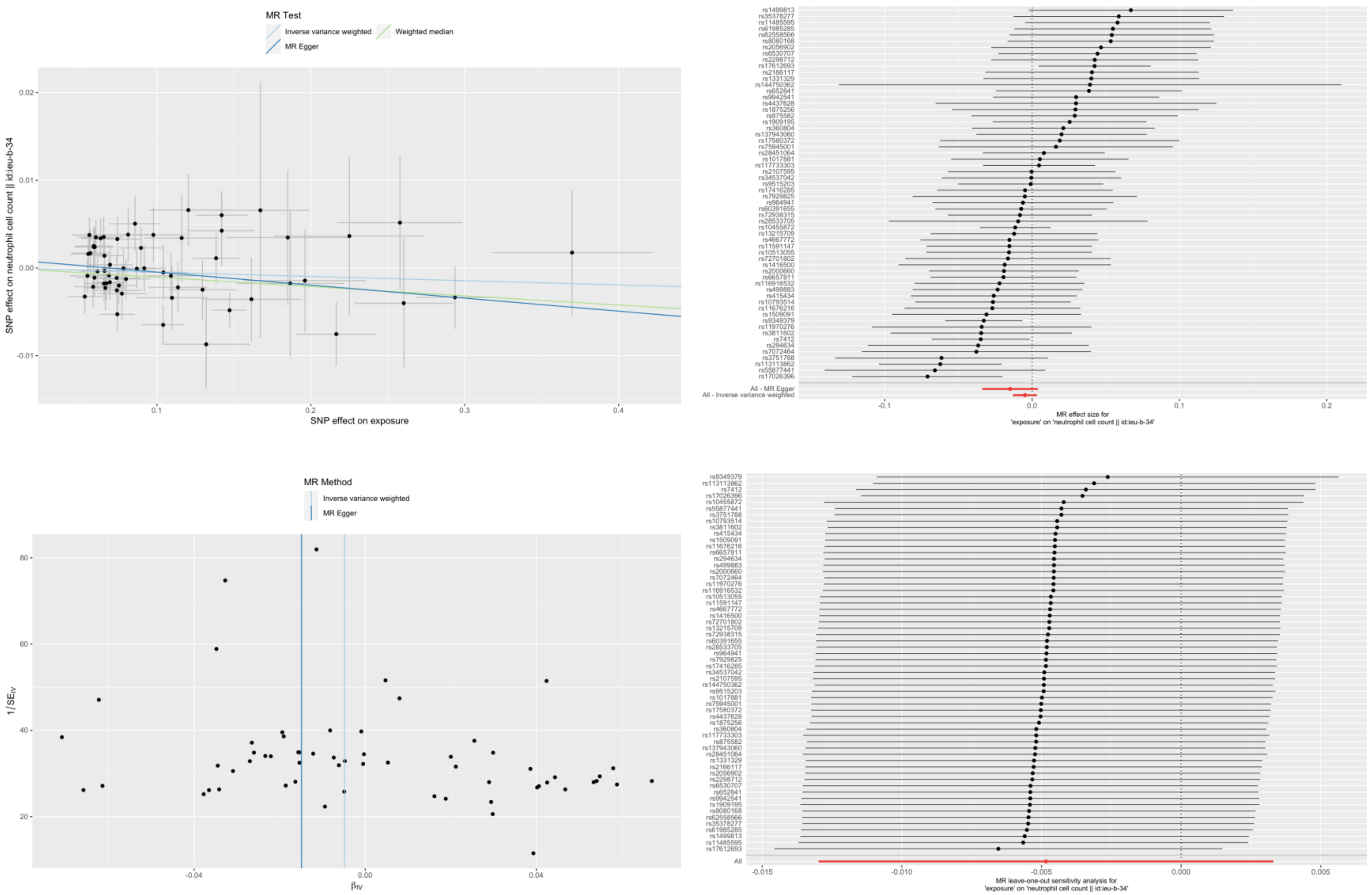

Supplemental Figure 16 Effect of myocardial infarction on peripheral neutrophil count

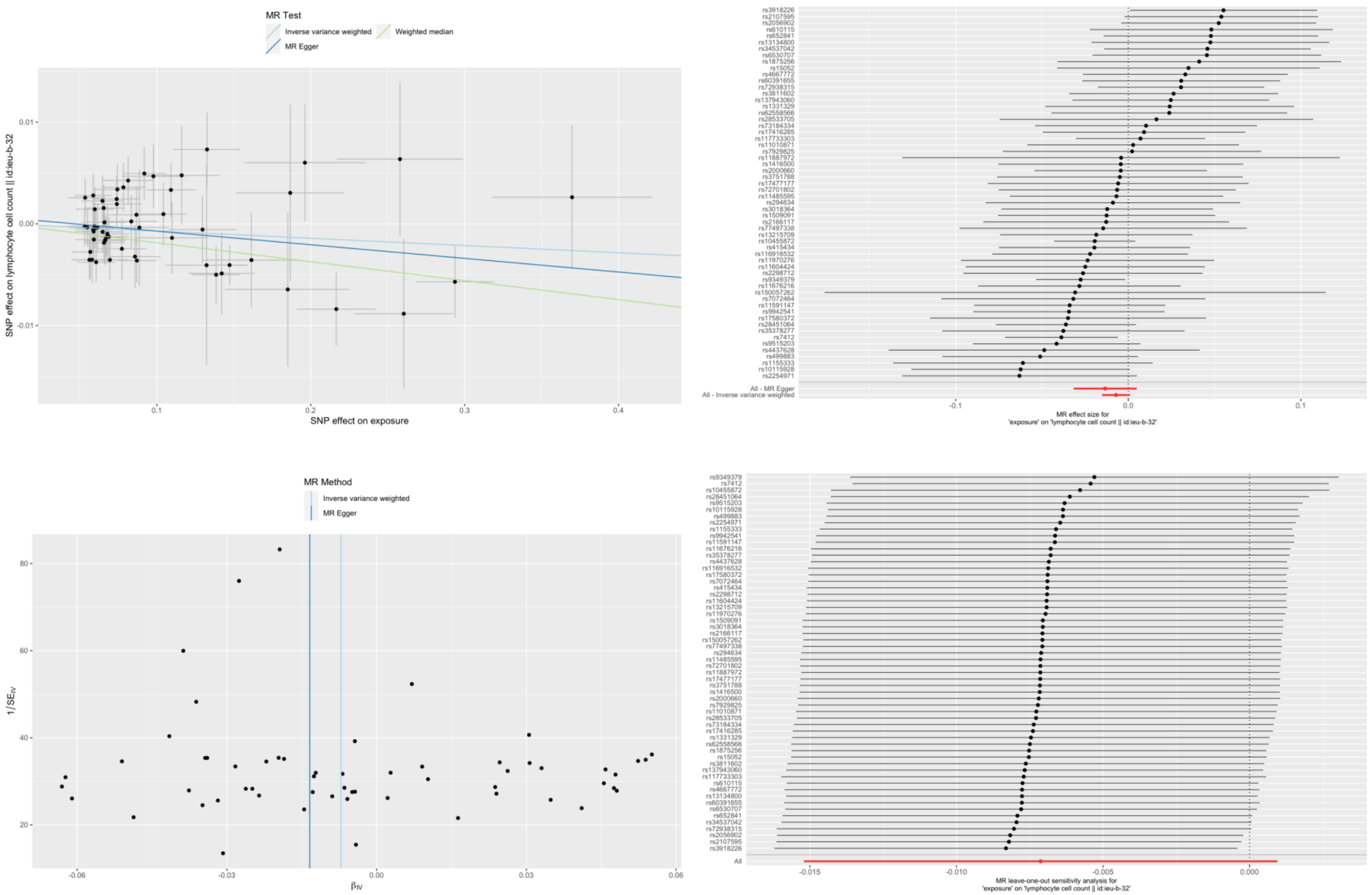

Supplemental Figure 17 Effect of myocardial infarction on peripheral lymphocyte count

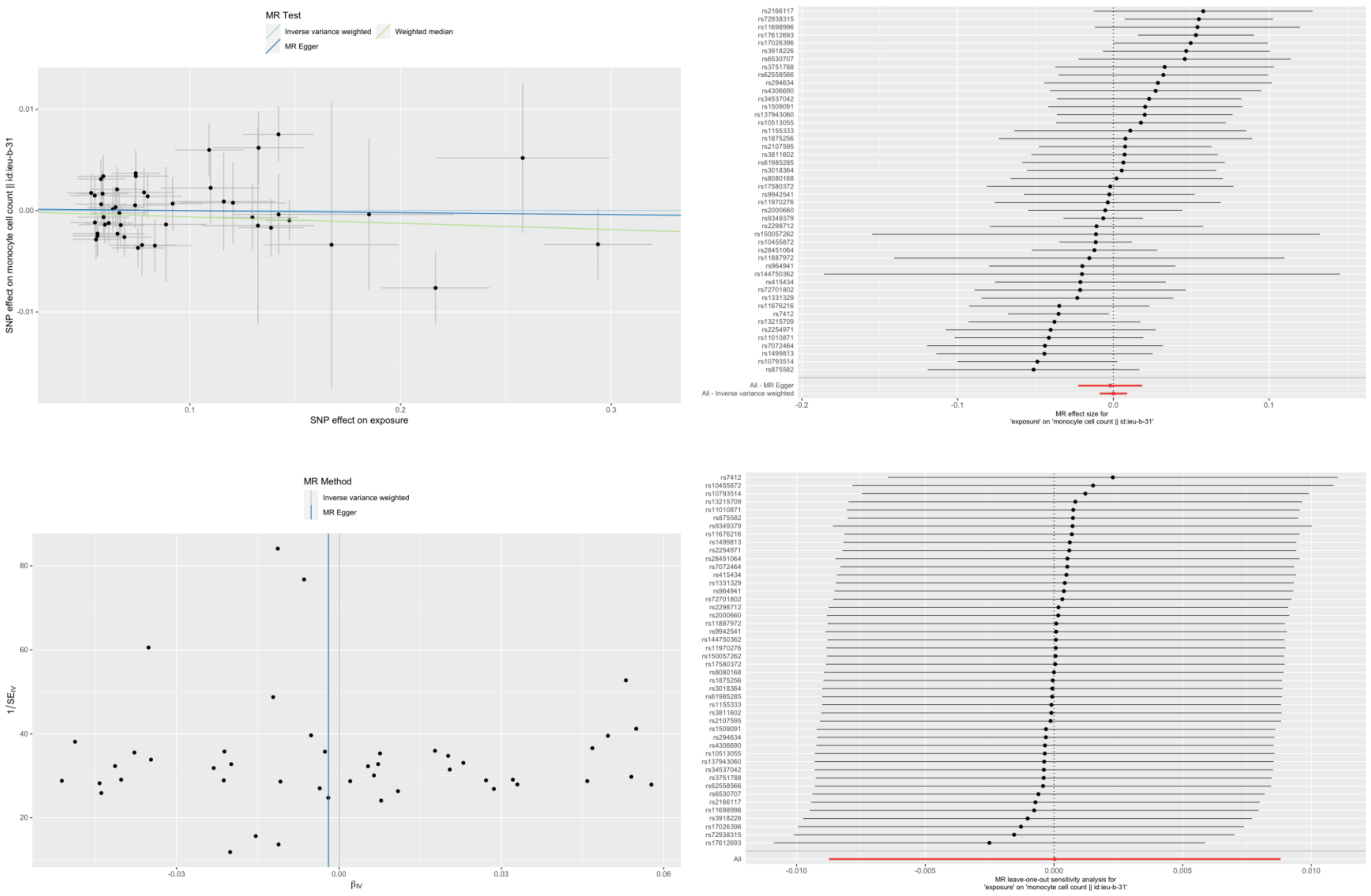

Supplemental Figure 18 Effect of myocardial infarction on peripheral mononuclear cell count
